# CSF sTREM2 is associated with neuroprotective microglial states early in Alzheimer's disease and deleterious effects later in the disease trajectory

**DOI:** 10.1002/alz.094051

**Published:** 2025-01-09

**Authors:** Harry Crook, Malak Wahdan, Marie Emilie Tuil, Nicholas R Livingston, Sanara Raza, Joseph Nowell, Paul Edison

**Affiliations:** ^1^ Imperial College London, London United Kingdom

## Abstract

**Background:**

Neuroinflammation is a key component of Alzheimer’s Disease (AD) pathology. Triggering receptor expressed on myeloid cells 2 (TREM2) is crucial to microglial involvement in AD, mediating trem2‐dependent activation and Disease‐Associated Microglia (DAM) polarization. However, GWAS revealed that loss‐of‐function mutations of its encoding gene are an important risk factor for AD. There is mounting evidence supporting a biphasic microglial response in AD trajectory, and such contrasting effects would support this. We evaluated the influence of cerebrospinal fluid soluble TREM2 (CSF sTREM2) on AD pathology in healthy, Mild Cognitive Impairment (MCI), and AD patients.

**Method:**

Patients were grouped by diagnosis and each group segmented according to amyloid and tau, into amyloid‐positive (Aß+) and negative (Aß‐), and tau‐positive (tau+) and negative (tau‐). We used a combination of CSF biomarkers and positron emission tomography (PET) scans to assess neuropathological substrates of AD: CSF p‐tau181 for tau pathology, CSF Amyloid‐ß 42 (Aß42) and 18F‐florbetapir (18F‐AV45) PET scans for amyloid deposition, and magnetic resonance imaging (MRI) scans for neurodegeneration.

**Result:**

We observed higher sTREM2 levels in Aß+/tau+ AD compared with Aß+/tau‐ AD and Aß‐/tau‐ MCI patients. Likewise, Aß+/tau+ MCI patients showed higher sTREM2 than Aß‐/tau‐MCI patients. Furthermore, sTREM2 levels were negatively associated with cortical volume in MCI and AD, and with MMSE scores in AD. sTREM2 was positively associated with CSF p‐tau181 in all groups except Aß+ controls. However, a positive association with CSF Aß42 was only observed in the Aß‐ control and the Aß+ and Aß‐ MCI groups. Similarly, sTREM2 was positively correlated with amyloid load (18F‐AV45 uptake) in controls and MCI patients yet negatively correlated in the AD group.

**Conclusion:**

The MCI and AD stages of the disease showed contrasting associations between sTREM2 and neuroimaging markers for AD pathology. These suggest diverging effects of the microglial response along AD trajectory, with a neuroprotective phase in early AD and a neurotoxic one later on. This could indicate that microglia polarize first towards an anti‐inflammatory phenotype before switching to a pro‐inflammatory one as neuropathology progresses.

